# Macular sensitivity and fixation patterns in normal eyes and eyes with uveitis with and without macular edema

**DOI:** 10.1007/s12348-011-0052-8

**Published:** 2011-12-14

**Authors:** Yasir J. Sepah, Elham Hatef, Elizabeth Colantuoni, Jianmin Wang, Mathew Shulman, Fatima Idrees Adhi, Abeer Akhtar, Mohamed Ibrahim, Afsheen Khwaja, Roomasa Channa, Raafay Sophie, Millena Bittencourt, Jangwon Heo, Diana V. Do, Quan Dong Nguyen

**Affiliations:** 1 Retinal Imaging Research and Reading Center, Wilmer Eye Institute, Johns Hopkins University School of Medicine, Baltimore, MD USA; 2 Bloomberg School of Public Health, Johns Hopkins University, Baltimore, MD USA; 3 Johns Hopkins Hospital, 600 North Wolfe Street Maumenee 745, Baltimore, MD 21287 USA

**Keywords:** Uveitis, Uveitic macular edema, Microperimetry, Retinal thickness

## Abstract

**Purpose:**

This study aims to investigate the relationship between macular sensitivity and thickness in eyes with uveitic macular edema (UME).

**Design:**

This study is a prospective observational case series.

**Methods:**

The setting for this study was clinical practice. The study included 59 (28 with UME, 31 without UME) eyes of 26 patients with uveitis and 19 eyes of 10 normal subjects. The procedure followed was fundus-related perimetry and retinal thickness map with an automated fundus perimetry/tomography system. Main outcome measures included quantification of macular sensitivity, fixation pattern, and relationship between macular sensitivity and thickness.

**Results:**

Fixation stability revealed that 56 eyes (93.44%) had stable fixation (>75% within the central 2° of point of fixation); three eyes (6.56%) were relatively unstable (<75% of fixation points located within 2°, >75% located within 4°); and no eye had unstable fixation (<75% of fixation points located within 4°). Evaluation of fixation location revealed that 45 eyes (76.27%) had central fixation location (>50% of fixation point within 0.5 mm of foveal center); seven eyes (11.86%) had peri-central fixation location (25% << 50% within 0.5 mm); and seven eyes (11.86%) had eccentric (<25% of fixation point within 0.5 mm) fixation location. We measured macular sensitivity and corresponding thickness in 1,708 loci of 61 study eyes. Macular sensitivity increased by 0.02 dB (95% confidence interval, 0.00, 0.06) per 1 μm increase in the thickness for the thickness values ≤280 μm. Macular sensitivity decreased by 0.04 dB (95% CI, −0.08, −0.01) per 1 μm increase in the thickness for the thickness values >280 μm.

**Conclusions:**

Perimetry quantification of macular sensitivity and retinal thickness, in association with other factors, may offer novel information regarding the impact of UME on retinal function.

## Introduction

Uveitis accounts for 300,000 new cases of legal blindness and 2.8–10% of all cases of blindness in the USA every year [1, 2]. Uveitis is known to cause a spectrum of morphological changes in the retina. Macular edema (ME) remains the leading cause of decreased vision in these patients and may be responsible for permanent visual impairment in 8.5% of the cases [3]. Although reversible in early stages, as the macular edema becomes chronic, it leads to permanent damage of the photoreceptor layer with progression to fibrosis [4]. Since chronic edema is a vision-threatening complication, the importance of effective monitoring strategies for uveitic macular edema (UME) becomes even more important.

Clinical suspicion of UME can be confirmed with the aid of a variety of investigations, including fluorescein angiography (FA), optical coherence tomography (OCT), and scanning laser ophthalmoscope (SLO), among others. FA has long been used to assess macular edema qualitatively. However, recently, OCT has been established to be of great value in the diagnosis and monitoring of macular edema, as it provides quantitative assessment of the retinal thickness at various locations [5]. Similarly, visual acuity (VA) is considered as the gold standard in assessing retinal function. However, change in retinal thickness resulting from the presence of ME does not necessarily correlate with the VA change in patients with UME [6]. Given that the functional outcome is the focus of interest and its improvement the objective of treatment for UME, such disconnect between VA and UME limits the use of OCT as a definitive tool to measure response to therapy. Moreover, central VA is not sufficient to fully characterize macular function [5, 7].

We investigate the effects of UME on macular functional parameters quantified by an automated microperimetry system [scanning laser ophthalmoscope/spectral domain optical coherence tomography (SLO/OCTTM)®, OPKO/OTI, Toronto, Canada] and correlate it with retinal thickness.

## Methods

The index prospective study was approved by the Johns Hopkins Medicine Institutional Review Board. Adult patients with uveitis who were evaluated at the Wilmer Eye Institute, Johns Hopkins University (Baltimore, MD, USA) were eligible for participation. The diagnosis of uveitis and macular edema was made by a uveitis specialist (QDN) using slit-lamp examination, contact lens biomicroscopy, and indirect ophthalmoscopy, and confirmed with optical coherence tomography (OCT), fundus photography, and fluorescein angiography (FA).

Additional study assessment included the Early Treatment Diabetic Retinopathy Study (ETDRS) best-corrected visual acuity (BCVA). The duration of uveitis, disease status (quiescent or active), presence of macular edema, and anatomical location of the disease (intermediate, posterior, or pan-uveitis), among others, were collected. Healthy adults with no known ocular diseases were enrolled in the study as controls. The OCT imaging and microperimetry were performed on healthy adults using same protocol as participants with uveitis.

OCT imaging was performed with the spectral-domain OCT module integrated in the OPKO/OTI® device. The spectral OCT/SLO module of the device generated three-dimensional (3D) retinal maps. It captured 28,000 A scans per second, which enabled the acquisition of up to 128 longitudinal OCT scans in 2s over a 5-mm area in the macula. The distance between the retinal nerve fiber layer and the hyporeflective line above the retinal pigment epithelium was measured automatically by the retinal thickness algorithm [8].

To perform microperimetry, a circular test pattern, the POLAR 3 (28 dots: four central, 12 mid, and 12 outer rings) was used for all patients. The following features were incorporated in the POLAR 3 pattern: Goldman-III stimulus size, 200-ms stimulus duration, and a 1,000-ms interval between stimuli presentation. Fundus localization based on retinal vessel alignment was automatically tracked by the spectral OCT/SLO system. The images from retinal topography and microperimetry were aligned, and 3D overlay images were created. For each of the 28 loci of the POLAR 3 test pattern, retinal thickness values were obtained. The paired data of microperimetry threshold and corresponding retinal thickness measurements were used to evaluate the relationship between retinal thickness and macular sensitivity [8].

The fixation pattern was evaluated as fixation location and fixation stability [9]. Fixation stability was classified into three categories: stable, relatively unstable, or unstable. If >75% of fixation points were located within a 2° diameter circle, regardless of the position of the foveal center, the fixation was classified as *stable*. If <75% of fixation points were located within a 2° circle, but >75% of fixation points were located within a 4°circle, fixation was classified as *relatively unstable*. If <75% of fixation points were located within a 4° circle, fixation was classified as *unstable*. Fixation location was documented in three categories: central, pericentral, and eccentric. If >50% of fixation points were within 0.5 mm of the foveal center, fixation was classified as *central*. If 25–50% of the fixation points were within 0.5 mm of the foveal center, fixation was classified as *pericentral*. If <25% of fixation points were within 0.5 mm of foveal center, fixation was classified as *eccentric*.

## Statistical analysis

### Uveitic macular edema

Exploratory data analyses included graphical (histograms, tables, side-by-side boxplots, and scatterplots) and statistical summaries (means, standard deviations, quantiles, and correlation coefficients) for (a) the distribution of each study variable, (b) the relationship between macular sensitivity and retinal thickness and potential confounding variables, and (3) the relationship between retinal thickness and potential confounding variables. The potential confounding variables include disease duration and status, underlying diseases and anatomical type of the disease, BCVA, fixation stability, fixation location, age, and gender. Age was treated as a categorical variable with levels: age <50, ≥50 to <70, and ≥70 years.

Linear mixed models were used to estimate the mean macular sensitivity as a function of retinal thickness adjusting for the potential confounding variables. The relationship between the mean macular sensitivity and retinal thickness was estimated separately for retinal thickness ≤350 and >350 μm; the cut-point of 350 μm was selected based on the exploratory data analysis.

There were several potential sources of correlation within the data set: measurements correlated within subjects, visits, eyes, and circular test pattern (central, mid-, and outer rings around the foveal center). Exploratory analysis of the potential sources of correlation was performed by fitting the mean model described above, assuming the data were independent and examining the residuals of that model for the various sources of correlation. The linear mixed model included random intercepts for subject, visits, eye, and circular test pattern [10].

Several sensitivity analyses were performed. The linear mixed model was fit, allowing the cut-point to vary from 340 to 370 μm. In addition, an analysis was performed to evaluate the effect of potential influential observations of retinal thickness by removing the upper and lower 1% of retinal thickness values from the data.

### Normal eyes and those with uveitis but no UME

With the same approach, the linear mixed models were used to estimate the mean macular sensitivity as a function of retinal thickness, BCVA, fixation stability, fixation location, age, gender, and ethnicity in eyes with uveitis and without macular edema comparing to healthy participants. The linear mixed model included random intercepts for subject, visits, eye, and circular test pattern. Age was treated as a categorical variable with levels: age <30, ≥30 to <40, ≥40 to <50, and ≥50 years. The relationship between the mean macular sensitivity and retinal thickness was estimated separately for retinal thickness ≤280 and >280 μm. To consider the effect of uveitis on the function of retina, an interaction term for disease presence (including those with macular edema and those without) and retinal thickness was considered in the model. The linear mixed model included random intercepts for subject, visits, and circular test pattern [10].

Several sensitivity analyses were performed. The linear mixed model was fit, allowing the cut-point to vary from 270 to 310 μm. In addition, an analysis was performed to evaluate the effect of potential influential observations of retinal thickness by removing the upper and lower 1% of retinal thickness values from the data.

Statistical analysis was performed using STATA version 10.1 (STATACORP, College Station, TX, USA). Statistical significance was reported if *p* < 0.05.

## Results

### Description of uveitis patients

A total of 26 patients (59 eyes) with the diagnosis of uveitis were enrolled in the study. Among these 26 patients, 14 patients contributed both eyes (28 eyes), and 12 patients contributed one eye at the initial visit, and eight patients (19 eyes) had follow-up visits. Tweny-eight eyes had UME, while 31 eyes with uveitis did not have macular edema. The age range was 16–86 years with a median of 45.50 years among the 26 patients. There were 15 women (57.69%) in our study. Of the total 26 patients, 11 (42%) had idiopathic posterior or pan-veitis, seven (26%) multifocal choroiditis, two (8%) punctate inner choroiditis, and additional two (8%) had birdshot choroidopathy. The remaining four (16%) patients had uveitis secondary to Lyme disease, sarcoidosis, autoimmune retinopathy, and ocular toxoplasmosis. Of type, uveitis was intermediate in nine (35%) patients and posterior in 14 (54%) patients; pan-uveitis was diagnosed in three (11%) of the patients. Disease duration at the time of microperimetry measurement ranged from 1 month to 27 years with median disease duration of 20 months. BCVA ranged from 20/12.5 to 20/500 in the study eye (median, 20/40). In 38 (65.52%) eyes, the uveitic disease was quiescent at the time of microperimetry measurement; 28 (47.46%) eyes had macular edema.

Of the total 59 examined uveitic eyes (28 with macular edema; 31 eyes without), 45 eyes (76.27%) had stable fixation; seven eyes (two eyes with ME and five without ME) (11.86%) had relatively unstable fixation; and seven eyes (all eyes without ME) (11.86%) had unstable fixation. The evaluation of fixation location revealed that 56 eyes (94.92%) had central fixation, and three eyes had (5.08%) peri-central fixation. There was no eye with eccentric fixation. The mean retinal thickness in an area within 3 mm of the foveal center was 311.03 μm [standard deviation (SD), 66.96 μm]; the mean macular sensitivity was 11.79 dB (SD, 4.98 dB).

### Description of normal participants

A total of 10 normal participants (19 eyes) were enrolled in the study. The age range was 25–63 years with a median of 42.5 years. There were seven women (70.0%) among the participants with no known ocular diseases. The normal participants and patients with uveitis were matched with regard to their age and gender distribution (*p* = 0.3270 and 0.4970, respectively, for the comparison of age and gender among two groups). BCVA ranged from 20/16 to 20/32 in the study eye (median, 20/20). Of the total 19 examined eyes, all had stable and central fixation. The mean retinal thickness in an area within 3 mm of the foveal center was 276.10 μm (SD, 33.57 μm), and the mean macular sensitivity was 16.48 dB (SD, 2.06 dB).

#### Macular sensitivity and retinal thickness in eyes with UME

**Fig. 1:**
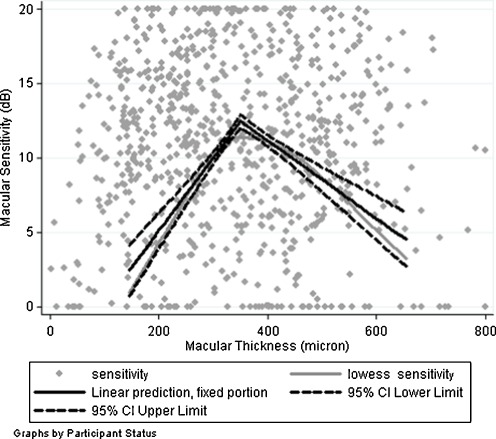
The relationship between the mean macular sensitivity as a function of retinal thickness in eyes with uveitic macular edema, as estimated by a LOWESS smooth function (*thick gray line*) as well as the fitted model for relationship between macular sensitivity and retinal thickness (*thick black line*) and 95% confidence interval (*dash lines*)

Macular sensitivity and corresponding retinal thickness were measured for 28 loci located on one of the three circular patterns of each of the 28 eyes with active macular edema at the time of microperimetry measurement, with a total number of 784 pair measurements of macular sensitivity and retinal thickness. The mean retinal thickness was 350.08 μm (SD, 69.33 μm), and the mean macular sensitivity was 10.61 dB (SD, 4.67 dB). Figure 1 displays the relationship between the mean macular sensitivity as a function of retinal thickness estimated by a locally weighted scatterplot smoothing (LOWESS) smooth function (thick gray line). At a thickness of 200 μm, the mean sensitivity was approximately 4 dB and then rises linearly to 11 dB by a thickness of 350 μm, but then decreases linearly to 6 dB by a thickness of 600 μm. It also presents the fitted model for relationship between macular sensitivity and retinal thickness (thick black line) as well as 95% CI (dash lines). Table 1 displays the estimated relationships between the macular sensitivity and retinal thickness as well as the potential confounders based on the linear mixed effects model. After removing the effects of retinal thickness and the potential confounders, approximately 4% of the variation in macular sensitivity could be attributed to differences across patients, and 31% of the variation could be attributed to differences between two eyes of each patient. An additional 3% of the variation could be explained by variation across different visits and microperimetry measurement on each eye; an additional 3% was due to variation across macular areas on three rings around the foveal center in each eye.

**Table 1:** Linear mixed effects model for the relationship among macular sensitivity, retinal thickness, and associated factors in eyes with uveitic macular edema

	Mean change in macular sensitivity (dB)	95% CI	*p* value
Retinal thickness ≤ 350 μm	0.02	0.01, 0.03	0.000
Retinal thickness > 350 μm	−0.02	−0.03, −0.01	0.000
Duration of uveitis (month)	0.08	0.00, 0.16	0.048
Disease status (active vs. stable)	4.62	0.66, 8.59	0.022
Underlying disease (other causes vs. idiopathic)	−2.45	−7.00, 2.10	0.291
Anatomical type (pan-uveitis vs. intermediate and posterior)	−5.84	−15.24, 3.57	0.224
Visual acuity (LOGMAR)	−1.64	−5.05, 1.77	0.346
Fixation stability (relatively unstable vs. stable)	−7.73	−13.42, −2.03	0.008
Fixation location (central, pericentral, eccenteric)	0.05	−2.19, 2.29	0.967
Age (>50 to ≤ 70 comparing to those ≤ 50)	−0.12	−4.42, 4.17	0.954
Age (>70 years comparing to those ≤ 50)	−1.26	−8.70, 6.19	0.741
Gender (male vs. female)	2.96	−1.40, 7.32	0.184

*CI* confidence interval, *dB* decibel, *DME* diabetic macular edema

After accounting for the potential confounding variables, the macular sensitivity increased by an average of 0.02 dB (95% CI, 0.01, 0.03) per 1 μm increase in the retinal thickness for the thickness values of 350 μm or less measured with the OPKO/OTI spectral-domain OCT. Macular sensitivity decreased by an average 0.02 dB (95% CI, −0.03, −0.01) per 1 μm increase in the thickness for thickness values of more than 350 μm. The estimated change in mean macular sensitivity for retinal thickness of 350 μm or less was different from the estimated change for values >350 μm (*p* < 0.0001).

The adjusted mean macular sensitivity decreased with age. Specifically, the adjusted mean macular sensitivity for patients 50–70 years of age was 0.12 dB smaller than those 50 years of age or younger (95% CI, −4.42, 4.17). The adjusted mean macular sensitivity among those older than 70 years was 1.26 dB smaller than the adjusted mean for patients of 50 years old or younger (95% CI, −8.70, 6.19).

Overall, the main findings were not sensitive to varying the cut-point (340–370 μm) for the association between mean macular sensitivity and retinal thickness. After removing the observations with the smallest and largest 1% of retinal thickness, the mean macular sensitivity was estimated to increase by 0.02 per 1 μm of retinal thickness for retinal thickness <350 μm, while macular sensitivity was estimated to decrease by 0.02 per 1 μm of retinal thickness for retinal thickness >350 μm.

#### Macular sensitivity in eyes with uveitis compared to normal participants

**Fig. 2:**
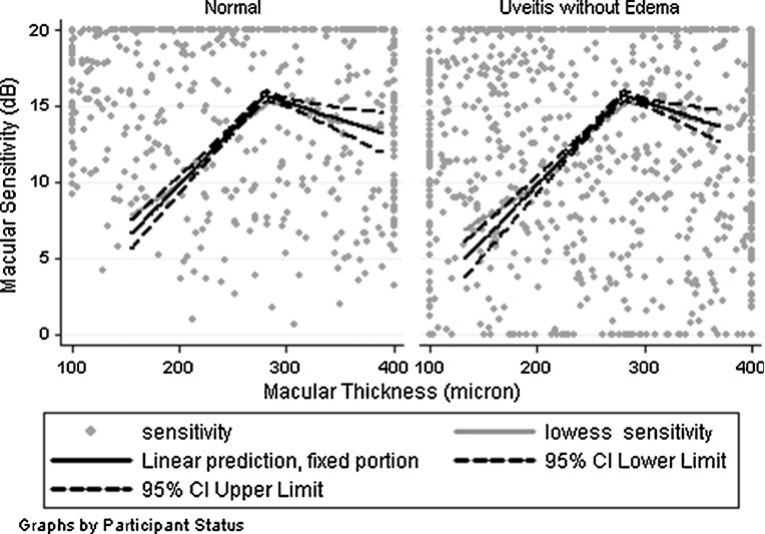
The relationship between the mean macular sensitivity as a function of retinal thickness in eyes with uveitis and eyes with no known ocular diseases, as estimated by a LOWESS smooth function (*thick gray line*) as well as the fitted model for relationship between macular sensitivity and retinal thickness (*thick black line*) and 95% confidence interval (*dash lines*)

Macular sensitivity and corresponding retinal thickness were measured for 28 loci located on one of the three circular patterns of each of the 31 eyes with a diagnosis of uveitis and no macular edema as well as 19 eyes of the normal participants, with a total number of 1,400 pair measurements of macular sensitivity and retinal thickness. Figure 2 displays the relationship between the mean macular sensitivity as a function of retinal thickness estimated by a LOWESS smooth function (thick gray line) for normal participants as well as those with uveitis and no macular edema. At a thickness of 200 μm, the mean sensitivity is roughly 10 dB in both groups, then rises linearly to 15 dB by a thickness of 280μm, then decreases linearly to 13 dB by a thickness of 380 μm. It also presents the fitted model for relationship between macular sensitivity and retinal thickness (thick black line) as well as 95% CI (dash lines). Table 2 displays the estimated relationships between the macular sensitivity and retinal thickness as well as the potential confounders based on the linear mixed effects model. After removing the effects of retinal thickness and the potential confounders, approximately 45% of the variation in macular sensitivity could be attributed to differences across participants. An additional 8% of the variation could be explained by variation across different visits and microperimetry measurement on each eye and an additional 7% by variation across macular areas on three rings around the foveal center in each eye. After accounting for the potential confounding variables, in the normal eyes, the macular sensitivity increased by an average of 0.02 dB (95% CI, 0.00, 0.03) per 1 μm increase in the retinal thickness for the thickness values of 280 μm or less measured with the OPKO/OTI spectral-domain OCT. The macular sensitivity had a slight change per 1 μm increase in the thickness for thickness values of more than 280 μm (mean, 0.00 dB, 95% CI, −0.02, 0.02). The estimated change in mean macular sensitivity for retinal thickness of 280 μm or less was different from the estimated change for values >280 μm (*p* < 0.0285).

**Table 2:** Linear mixed effects model for the relationship among macular sensitivity, retinal thickness, and associated factors in eyes with uveitis as well as normal participants

	Mean Change in Macular Sensitivity (dB)	95% CI	*p* value
Uveitis vs. normal eyes	−1.87	−5.03, 1.29	0.246
Normal participants, retinal thickness ≤ 280 μm	0.02	0.00, 0.03	0.015
Normal participants, retinal thickness > 280 μm	0.00	−0.02, 0.02	0.749
Uveitis patients, retinal thickness ≤ 280 μm	0.04	0.03, 0.05	0.000
Uveitis patients retinal thickness > 280 μm	−0.04	−0.07, −0.02	0.000
Visual acuity (LOGMAR)	−0.41	−2.48, 1.66	0.698
Fixation stability (stable, relatively unstable, unstable)	−0.05	−1.57, 1.47	0.951
Fixation oocation (central, pericentral, eccentric)	0.86	0.19, 1.54	0.012
Age (≥ 30 to < 40 comparing to those < 30)	1.34	−2.62, 5.31	0.507
Age (≥ 40 to < 50 comparing to those < 30)	1.55	−2.25, 5.35	0.424
Age (≥50 years comparing to those < 30)	−2.50	−6.57, 1.57	0.229
Gender (male vs. female)	0.70	−2.08, 3.47	0.622
Ethnicity (Caucasian, African American, Asian, Hispanic, unknown)	0.22	−1.29, 1.74	0.772

*CI* confidence interval, *dB* decibel

In patients with uveitis and no macular edema, the macular sensitivity increased by an average of 0.04 dB (95% CI, 0.03, 0.05) per 1 μm increase in the retinal thickness for the thickness values of 280 μm or less. The macular sensitivity decreased by an average of 0.04 dB (95% CI, −0.07, −0.02) per 1 μm increase in the thickness for thickness values of more than 280 μm. The adjusted mean macular sensitivity changed with age. Specifically, the adjusted mean macular sensitivity for patients 30–40 years of age and 40–50 years of age was 1.34 and 1.55 dB different from those 30 years of age (95% CI, −2.62, 5.31 and −2.25, 5.35, respectively). The adjusted mean macular sensitivity among those 50 years or older was 2.50 dB smaller than the adjusted mean for patients <50 years old (95% CI, −6.57, 1.57).

The main findings were not sensitive to varying the cut-point (270–310 μm) for the association between mean macular sensitivity and retinal thickness. After removing the observations with the smallest and largest 1% of retinal thickness, the mean macular sensitivity was estimated to increase by 0.02 and 0.04 per 1 μm of retinal thickness for retinal thickness <280 μm (among normal participants and patients with uveitis). The estimated association for retinal thickness >280 μm changed very little in both treatment groups.

## Discussion

Morbidity in eyes with uveitis often results from chronic and recurrent episodes of inflammation, which are associated with duration of each episode, frequency of attacks, and anatomic location of uveitis [11]. Such repeated attacks may cause cumulative damage that may, over time, lead to irreversible tissue damage. In order to avoid cumulative damage of the disease, it is important that the inflammation is managed aggressively [12] and the retinal function of patients is monitored objectively [13]. Microperimetry provides us with the necessary tools and may prove to be an integral part in the management of uveitis patients.

Microperimetry (or fundus-related perimetry) assesses macular sensitivity and provides an almost exact correlation between fundus disease and corresponding functional defects while taking into account the fixation pattern and stability [14, 15]. Microperimetry (MP) testing has been previously available in other devices, such as the Rodenstock® SLO and the automatic fundus-related perimeter, (MP1 Microperimeter®; Nidek Technologies, Tokyo, Japan). These devices have been used to evaluate various retinal conditions such as diabetic macular edema, age-related macular degeneration, idiopathic macular telengectasia, and central serous retinopathy, using different measurement schemes [7, 14–17]. In the current study, we measured the sensitivity using the OPKO/OTI® device, an automated fundus perimetry/tomography system, with a microperimetric radial pattern to cover central 12° of the macula in eyes with uveitis and UME. The advantages of using OPKO/OTI® device include simultaneous SD-OCT, better image quality, and readily accessible structural and functional correlation of the 28 loci measured. Both MP-1 and OPKO/OTI devices are capable of performing serial examinations; however, the latter requires much less time to complete the exam [18]. The OPKOP/OTI® device has been employed to evaluate eyes with DME [19] and has shown consistent results.

Although the correlation between retinal thickness and visual acuity is controversial, patients with UME have been previously demonstrated to have a negative correlation between macular sensitivity and retinal thickness. Roesel et al. [6] studied 31 uveitis patients (53 eyes) and concluded that with increase in retinal thickness, the retinal sensitivity decreases. Lardenoye et al. [20] also reported decreased sensitivity in the macular area of patients with edema, though they only had five patients with inflammatory edema included in the study. Other investigators studying diabetic patients have also reported a decrease in retinal sensitivity when ME develops and its deterioration in eyes at more severe stages of ME compared to the normal population [19, 21–25].

The results of the current study are in partial agreement with what has already been reported in the literature. We observed that in the eyes with UME, there was a slight increase in the retinal sensitivity with each 1 μm increase in retinal thickness when the thickness value was ≤350 μm. A decrease in retinal sensitivity was seen with every 1 μm increase in thickness thereafter. A similar trend was noted in uveitic eyes without ME and eyes with no known ocular diseases. However, in these two groups of patients, the thickness value beyond which we observed a decline in retinal sensitivity was 280 μm, 100 μm less than what was seen in UME eyes. Landa et al. [18] also found a decrease in retinal sensitivity with thinning of the retina. One possible explanation for this variation may be a stress response, which results in the emergence of a hypersensitive retina as a consequence to the mechanical stress imparted by fluid accumulation in addition to the inflammatory insults to the tissue. Such stress responses have been well documented in cardiac and pancreatic tissue.

Uveitic eyes without ME not only had lower retinal sensitivity compared to normal eyes, but the corresponding rise in retinal sensitivity with each rise in retinal thickness was more in normal eyes compared to eyes with uveitis. Although the decrease in sensitivity in uveitic eyes without ME was not statistically significant (*p* = 0.246), it may indicate that macular edema may not be the only factor affecting retinal sensitivity and that the inflammatory process in eyes with uveitis may affect retinal sensitivity. The cut points of 350 μm in eyes with UME and 280 μm in uveitic eyes without ME were chosen based on the exploratory data analysis. Unadjusted factors in the model, such as the frequency of flare-ups, duration of ME, and duration of disease, may play a role in the relationship between macular sensitivity and thickness. There may be the possibility that areas with higher macular thickness and edema have active disease compared to areas with normal thickness retina. Retinal thinning or atrophy either due to the development of cystic changes or loss of photoreceptors have been shown to decrease retinal sensitivity as well [26, 27]. It should be noted that differences in measuring devices (OPKO/OTI® vs MP-1®) and methods of data analysis may also result in variation in results.

In the current study, fixation was reported to be located centrally in majority of the cases (94.95%). We did not find any significant correlation between fixation location and retinal sensitivity even after adjusting for confounding factors (age, retinal thickness, duration of disease, etc.). Roesel et al. [6] had noted similar findings in UME patients and found that fixation was centrally located in 79% of the cases, relatively eccentric in 14%, and predominantly eccentric in only 7%. Although we had a small number of eyes with unstable fixation (relative and predominant), our model was able to detect a significant (*p* = 0.008) decline in the sensitivity of the retina in these eyes. Roesel et al. [6]. showed similar findings in UME patients, pertaining to correlation of fixation location/stability with retinal thickness and sensitivity, but they found that fixation was stable only in 47% and was eccentric in 21% of the UME eyes. These differences in the location of fixation can be attributed to differences among the two study populations. Our study population was younger (median = 45 years), while that of Roesel et al. was older (mean = 51, SD ± 14). Furthermore, 70% of the population in Roesel’s study also had an epiretinal membrane at the time of the study. Retinal thickness was significantly higher (*p* = 0.003), and VA was significantly lower (*p* = 0.08) in these patients compared to the subjects in our study. Another reason could be the fact that we confirmed the presence of macular edema in all of our patients with spectral domain OCT in conjunction with FA, while Roesel et al. had used only FA for the detection and confirmation of ME. There may be cases of macular edema that do not demonstrate significant leakage on FA for diagnosis. Our results are consistent with findings by Vujosevic et al. [28] who investigated retinal fixation impairments in DME patients.

We also noted that there was a small but statistically significant increase in the sensitivity of the retina with an increase in the duration of disease. Such an increase in the sensitivity with disease duration may be because of the time required by the retina to recover from the initial insults, and as time passes by, the patient receives adequate therapy and the retinal sensitivity recovers. White and Bedell did show that patients with longer duration since onset of (macular disease) show oculomotor behaviors qualitatively more like those of normal eyes [29]. Other findings in our study suggest a trend of decrease in retinal sensitivity with age both in eyes with UME and eyes without ME.

There are several limitations in our study. In addition to a small study sample size, we were not able to adjust for certain factors in our model such as the number of recurrences of ME, number of disease flare-ups, duration of UME and uveitis, and previous medical intervention, as well as other ophthalmic conditions such as refractive error. The recurrent episodes of UME as well as flare-ups of inflammation might induce a gradual decrease in the sensitivity and changes in the normal anatomy of the retina, which affects the relationship between retinal sensitivity and thickness. Similarly, retinal sensitivity may also be affected by the duration of each flare-up or episode of UME.

In conclusion, the results from our study have provided a novel insight into the relationship between fixation, retinal thickness, and retinal sensitivity among patients with uveitis, with and without macular edema. We recognize that additional, larger studies are required to further explore these relationships and to enhance our understanding of macular function in ocular inflammatory diseases, and look forward to contributing to such investigations.

## References

[1] Rothova A (1996). Causes and frequency of blindness in patients with intraocular inflammatory disease. Br J Ophthalmol.

[2] Suttorp-Schulten MS, Rothova A (1996). The possible impact of uveitis in blindness: a literature survey. Br J Ophthalmol.

[3] Malinowski SM, Pulido JS, Folk JC (1993). Long-term visual outcome and complications associated with pars planitis. Ophthalmology.

[4] Gallagher MJ (2007). The characteristic features of optical coherence tomography in posterior uveitis. Br J Ophthalmol.

[5] Sakata LM (2009). Optical coherence tomography of the retina and optic nerve—a review. Clin Experiment Ophthalmol.

[6] Roesel M (2011). Comparison of retinal thickness and fundus-related microperimetry with visual acuity in uveitic macular oedema. Acta Ophthalmol.

[7] Rohrschneider K, Bultmann S, Springer C (2008). Use of fundus perimetry (microperimetry) to quantify macular sensitivity. Progress in Retinal and Eye Research.

[8] Landa G (2010). Combined three-dimensional spectral OCT/SLO topography and microperimetry: steps toward achieving functional spectral OCT/SLO. Ophthalmic Res.

[9] Fujii GY (2003). Characteristics of visual loss by scanning laser ophthalmoscope microperimetry in eyes with subfoveal choroidal neovascularization secondary to age-related macular degeneration. Am J Ophthalmol.

[10] Kung-Yee Liang PH, Peter Diggle J, Scott Zeger L (2002). Analysis of longitudinal data.

[11] Durrani OM (2004). Degree, duration, and causes of visual loss in uveitis. Br J Ophthalmol.

[12] Smith JR (2004). Management of uveitis. Clinical and Experimental Medicine.

[13] Nguyen QD et al (2006) Treating chronic noninfectious posterior segment uveitis: the impact of cumulative damage. Proceedings of an expert panel roundtable discussion. Retina (Suppl):1–16 10.1097/01.iae.0000250601.15893.5f 17050954

[14] Toonen F (1995). Microperimetry in patients with central serous retinopathy. Ger J Ophthalmol.

[15] Carpineto P (2007). Fundus microperimetry patterns of fixation in type 2 diabetic patients with diffuse macular edema. Retina.

[16] Charbel Issa P (2008). Correlation of macular function with retinal thickness in nonproliferative type 2 idiopathic macular telangiectasia. Am J Ophthalmol.

[17] Ojima Y (2008). Retinal sensitivity measured with the micro perimeter 1 after resolution of central serous chorioretinopathy. Am J Ophthalmol.

[18] Landa G (2010). Combined three-dimensional spectral OCT/SLO topography and microperimetry: steps toward achieving functional spectral OCT/SLO. Ophthalmic Research.

[19] Hatef ECE, Wang J, Ibrahim M, Shulman M, Adhi F, Sepah YJ, Channa R, Khwaja A, Nguyen QD, Do DV (2011). The relationship between macular sensitivity and retinal thickness in eyes with diabetic macular edema. Am J Ophthalmol.

[20] Lardenoye CWTA, van Kooij B, Rothova A (2006). Impact of macular edema on visual acuity in uveitis. Ophthalmology.

[21] Vujosevic S (2006). Diabetic macular edema: correlation between microperimetry and optical coherence tomography findings. Invest Ophthalmol Vis Sci.

[22] Kube T (2005). Fixation stability and macular light sensitivity in patients with diabetic maculopathy: a microperimetric study with a scanning laser ophthalmoscope. Ophthalmologica.

[23] Okada K (2006). Correlation of retinal sensitivity measured with fundus-related microperimetry to visual acuity and retinal thickness in eyes with diabetic macular edema. Eye (Lond).

[24] Shah VA, Chalam KV (2006). Letter regarding correlation of retinal sensitivity measured with fundus related microperimetry to visual acuity and retinal thickness in eyes with diabetic macular oedema. Eye (Lond).

[25] Rohrschneider K (2000). Scanning laser ophthalmoscope fundus perimetry before and after laser photocoagulation for clinically significant diabetic macular edema. Am J Ophthalmol.

[26] Crossland MD (2005). Preferred retinal locus development in patients with macular disease. Ophthalmology.

[27] Crossland MD, Culham LE, Rubin GS (2004). Fixation stability and reading speed in patients with newly developed macular disease. Ophthalmic Physiol Opt.

[28] Vujosevic S (2006). Diabetic macular edema: correlation between microperimetry and optical coherence tomography findings. Investig Ophthalmol Vis Sci.

[29] White JM, Bedell HE (1990). The oculomotor reference in humans with bilateral macular disease. Invest Ophthalmol Vis Sci.

